# Effects of a LPG stove and fuel intervention on adverse maternal outcomes: A multi-country randomized controlled trial conducted by the Household Air Pollution Intervention Network (HAPIN)

**DOI:** 10.1016/j.envint.2023.108059

**Published:** 2023-08

**Authors:** Ashley Younger, Abbey Alkon, Kristen Harknett, Miles A. Kirby, Lisa Elon, Amy E. Lovvorn, Jiantong Wang, Wenlu Ye, Anaité Diaz-Artiga, John P. McCracken, Adly Castañaza Gonzalez, Libny Monroy Alarcon, Alexie Mukeshimana, Ghislaine Rosa, Marilu Chiang, Kalpana Balakrishnan, Sarada S. Garg, Ajay Pillarisetti, Ricardo Piedrahita, Michael Johnson, Rachel Craik, Aris T. Papageorghiou, Ashley Toenjes, Ashlinn Quinn, Kendra N. Williams, Lindsay Underhill, Howard H. Chang, Luke P. Naeher, Joshua Rosenthal, William Checkley, Jennifer L. Peel, Thomas F. Clasen, Lisa M. Thompson, HAPIN investigators

**Affiliations:** a School of Nursing, University of California, San Francisco, San Francisco, CA, USA; b Department of Global Health and Population, Harvard T. H. Chan School of Public Health, Harvard University, Boston, MA, USA; c Department of Biostatistics and Bioinformatics, Rollins School of Public Health, Emory University, Atlanta, GA, USA; d Gangarosa Department of Environmental Health, Rollins School of Public Health, Emory University, Atlanta, GA, USA; e Division of Environmental Health Sciences, School of Public Health, University of California, Berkeley, CA, USA; f Center for Health Studies, Universidad del Valle de Guatemala, Guatemala City, Guatemala; g Department of Environmental Health Science, College of Public Health, University of Georgia, Athens, GA, USA; h Eagle Research Centre, Kigali, Rwanda; i Department of Disease Control, London School of Hygiene and Tropical Medicine, London, UK; p Center for Global Non-Communicable Disease Research and Training, Johns Hopkins University, Baltimore MD, USA; j Department of Environmental Health Engineering, ICMR Center for Advanced Research on Air Quality, Climate and Health, Sri Ramachandra Institute for Higher Education and Research (Deemed University), Chennai, India; k Berkeley Air Monitoring Group, Berkeley, CA, USA; l Nuffield Department of Women’s and Reproductive Health, University of Oxford, Oxford, UK; m Cardiovascular Division, Department of Medicine, Washington University in St. Louis, St. Louis, MO, USA; o Center for Global Non-Communicable Disease Research and Training, School of Medicine, Johns Hopkins University, Baltimore, MD, USA; n Division of Epidemiology and Population Studies, Fogarty International Center, National Institutes of Health, Bethesda, MD, USA, Division of Pulmonary and Critical Care, Johns Hopkins University School of Medicine, Baltimore, MD, USA; q Department of Environmental and Radiological Health Sciences, Colorado State University, Fort Collins, CO, USA; r Nell Hodgson Woodruff School of Nursing, Emory University, Atlanta, GA, USA

**Keywords:** Cooking fuel, Birth outcomes, Household air pollution, Low- and middle-income countries, Spontaneous abortion, Hypertensive disorders of pregnancy, Postpartum hemorrhage and maternal mortality

## Abstract

Household air pollution from solid cooking fuel use during gestation has been associated with adverse pregnancy and birth outcomes. The Household Air Pollution Intervention Network (HAPIN) trial was a randomized controlled trial of free liquefied petroleum gas (LPG) stoves and fuel in Guatemala, Peru, India, and Rwanda. A primary outcome of the main trial was to report the effects of the intervention on infant birth weight. Here we evaluate the effects of a LPG stove and fuel intervention during pregnancy on spontaneous abortion, postpartum hemorrhage, hypertensive disorders of pregnancy, and maternal mortality compared to women who continued to use solid cooking fuels. Pregnant women (18–34 years of age; gestation confirmed by ultrasound at 9–19 weeks) were randomly assigned to an intervention (n = 1593) or control (n = 1607) arm. Intention-to-treat analyses compared outcomes between the two arms using log-binomial models. Among the 3195 pregnant women in the study, there were 10 spontaneous abortions (7 intervention, 3 control), 93 hypertensive disorders of pregnancy (47 intervention, 46 control), 11 post postpartum hemorrhage (5 intervention, 6 control) and 4 maternal deaths (3 intervention, 1 control). Compared to the control arm, the relative risk of spontaneous abortion among women randomized to the intervention was 2.32 (95% confidence interval (CI): 0.60, 8.96), hypertensive disorders of pregnancy 1.02 (95% CI: 0.68, 1.52), postpartum hemorrhage 0.83 (95% CI: 0.25, 2.71) and 2.98 (95% CI: 0.31, 28.66) for maternal mortality. In this study, we found that adverse maternal outcomes did not differ based on randomized stove type across four country research sites.

## 1 Introduction

Approximately 2.3 million premature deaths and 91.5 million disability-adjusted life years (DALYs) were attributed to household air pollution globally annually (Bennitt et al., 2021). Household air pollution has been associated with asthma, acute respiratory infection, chronic obstructive pulmonary disease, lung cancer, cerebrovascular and ischemic heart disease, low birthweight, stillbirth, under-5 mortality, as well as respiratory and cardiovascular mortality (Bennitt et al., 2021, Lee et al., 2020). The burden of adverse health effects attributed to household air pollution mainly occurs in low- and middle-income countries (LMICs) where polluting fuels and technologies are often used for cooking. The incomplete combustion of solid cooking fuels (e.g., wood, coal, crop residue, animal dung) releases pollutants such as carbon monoxide (CO) and particulate matter (PM) (Smith et al., 2014). Fine particulate matter (particles with a diameter of ≤ 2.5 μm [PM_2_._5_) can penetrate the lungs and cross into the blood stream increasing the risk for a wide range of diseases. In 2013 the World Health Organization (WHO) and the International Agency for Research on Cancer (IARC) classified PM as carcinogenic to humans (WHO, 2013). In 2021, the WHO reduced their Global Air Quality Guidelines (AQGs) for annual ambient mean concentrations for PM_2.5_ from 10 µg/m^3^ to 5 μg/m^3^ (WHO, 2021). Oxidative stress, DNA methylation and mitochondrial DNA alteration, and endocrine disruption have been identified as potential mechanisms for PM_2.5-_induced adverse health effects (Li et al., 2019). Pollutants released from cooking with solid fuels such as PM_2.5_ and CO can be absorbed into the maternal blood stream increasing risk of adverse health effects and potentially affecting fetal growth by directly crossing the placenta (Amegah et al., 2014, Dutta, 2017). The Ghana Randomized Air Pollution and Health Study (GRAPHS) randomized 1414 pregnant women into 3 groups: liquified petroleum gas (LPG) cookstove, improved biomass cookstove or traditional 3-stone fire (control group). Participants were monitored for 72-hr personal CO exposure and cord blood was collected immediately following delivery. The study demonstrated a decreased biomarker of mitochondrial function that reflects cumulative oxidative stress markers in cord blood of children in the control group compared to the clean cookstove intervention groups (Kaali et al., 2018), whereas the LPG stove intervention increased the mitochondrial function biomarker and therefore reduced cumulative oxidative stress markers compared to the improved biomass stove and the 3-stone fires. CO also has the ability to cross the placental barrier and compromise oxygen delivery to fetal circulation since fetal hemoglobin has a higher affinity to CO than adult hemoglobin (Amegah et al., 2014, Di Cera et al., 1989, Longo, 1977).

Several studies have linked household air pollution exposure with adverse birth outcomes like low birth weight, stillbirth, and neonatal death, though evidence on maternal health outcomes has been mixed (Amegah et al., 2014, Alexander et al., 2018, Agrawal and Yamamoto, 2015, Mukherjee et al., 2015, Samaraweera and Abeysena, 2010, Weber et al., 2020). The quality of the evidence from prior research between polluting fuel use on pregnancy outcomes is limited by variability in exposure assessment, residual and unmeasured confounding, and inconsistency in assessing birth outcomes (Younger et al., 2022).

The Household Air Pollution Intervention Network (HAPIN) Trial was a randomized controlled trial (RCT) of a LPG stove intervention with continuous, free fuel distribution and behavioral reinforcement to encourage LPG use in intervention homes across four countries: Guatemala, India, Peru and Rwanda. The overall objective of the HAPIN trial was to investigate the effect of a randomized LPG stove and fuel intervention on health outcomes in four diverse LMIC populations, determine the exposure–response relationships and measure targeted and exploratory biomarkers of exposure on health effects (Barr et al., 2020, Johnson, et al., 2021, Ye, 2022). In the HAPIN trial, the mean difference in newborn birth weight was 19.6 g (95% confidence interval, −10.1 to 49.2) for infants in the intervention arm compared to those in the control arm (n = 3061 live births) (Clasen et al., 2022). There was a 66% mean reduction in exposure to particulate matter with an aerodynamic diameter ≤ 2.5 µm (PM_2.5_) and a 70% reduction in black carbon (BC) among pregnant women in the intervention arm compared to the controls. An inter-quartile increase in average prenatal exposure to PM_2.5_ (74.5 µg/m3) had no effect on birthweight, while an interquartile increase in BC (7.3 ug/m3) was associated with a decrease in birthweight (21·9g (95% confidence interval, −37.3 to −6.1) (Balakrishnan, et al., 2022, Johnson, et al., 2021).

The objective of this study is to investigate whether adverse maternal outcomes (spontaneous abortion, hypertensive disorders of pregnancy, postpartum hemorrhage and maternal mortality) differ based on study arm across four country sites within the HAPIN trial. Given that the LPG intervention reduced personal exposures to household air pollution during pregnancy on average, we hypothesize that it would also reduce the incidence of adverse maternal outcomes compared to women in the control arm who continued to cook with solid fuels during pregnancy.

## 2 Materials and methods

### 2.1 Trial design and study settings

The HAPIN trial was a randomized controlled trial of LPG stoves and free fuel distribution conducted in 3200 households across four low-and middle-income countries with primarily rural study settings with a range of elevations: India (Tamil Nadu, 2 study sites), Guatemala (Jalapa municipality, 1 study site) Peru (Department of Puno, 6 study sites) and Rwanda (Eastern Province, 1 study site). In an effort to increase generalizability, the study sites were chosen to represent diverse characteristics from altitude, cooking practices, and baseline pollution levels. The mean travel time to the nearest health facility from any location in the study areas ranged from 15 to 48 min (Simkovich et al., 2022). The trial utilized stove and fuel delivery records, questionnaires, visual observations and temperature-logging sensors that continuously monitored traditional stoves in intervention homes throughout the trial to evaluate fidelity and adherence to LPG stove use (Johnson et al., 2020, Wilson et al., 2020). Education and behavior reinforcement for exclusive LPG stove use occurred in intervention arm homes (Williams et al., 2020, Wilson et al., 2020). Results showed 96% of participants in the LPG intervention arm reported cooking exclusively with LPG at the two follow-up visits during the prenatal period. Traditional stove use was detected via SUMs on a median (interquartile range) of 0.0% (0.0%, 1.6%) of follow-up days (median follow-up = 134 days) (Quinn et al., 2021). The study protocol is available at ClinicalTrials.gov Identifier: NCT02944682 and study methods are detailed in the study design paper (Clasen et al., 2020).

### 2.2 Participant recruitment and enrollment

Eligible pregnant women were identified at antenatal clinics and enrolled over a 23-month period (April 2018 to February 2020) in cooperation with local ministries of health (MoH) and health systems personnel. Eight hundred pregnant women who used traditional biomass stoves for cooking were enrolled from each country (aged 18 to 34 years, 9 to 19 weeks gestation confirmed by ultrasound) (Clasen et al., 2020). Participants were randomly assigned to receive a LPG cookstove and a supply of free fuel delivery (intervention arm) or to the control arm, with the expectation that they would continue cooking with a biomass cookstove. Informed consent was obtained from all study participants that met eligibility requirements, and participants were able to withdraw from the study at any time.

### 2.3 Randomization and intervention

Randomization lists were assembled by the Emory University data management core and assignments were sent to the participating international research centers in sealed tamper-proof envelopes. Assignments were stratified into two sites in India and six sites in Peru. Eligible participants were randomized into intervention and control arms by selecting one of six envelopes presented to them by trained study field staff visiting their homes. The intervention households received a locally available LPG stove as well as continuous supply and delivery of free LPG fuel for 18 months during the study. Exclusive stove use during fuel deliveries was encouraged and reinforced by field staff and details of the behavior change program have been previously published (Williams et al., 2020).

### 2.4 Data collection and outcome measures

A total of 3200 pregnant women were randomized. Following recruitment and informed consent, a baseline survey was administered by a trained, local field staff member. The survey included questions about cooking behaviors, household characteristics, socioeconomic and demographic information, medical and obstetric history, physical activity, dietary diversity, and household food insecurity. The resting blood pressure of enrolled pregnant women was measured in triplicate; height and weight was measured in duplicate (Clasen et al., 2020). Personal exposure to PM_2.5_, black carbon (BC), and carbon monoxide (CO) were measured three times during pregnancy for 24-hours: once at baseline (pre-randomization, 9 to < 20 weeks of gestation) and twice post-randomization at 24 – 28 weeks and 32 – 36 weeks of gestation. Personal PM_2.5_ exposure was measured using the Enhanced Children’s MicroPEM^TM^ (ECM, RTI International, Research Triangle Park, NC). BC exposure was measured on sampled PM2.5 filters using a SootScan™ Model OT21 transmissometer (Magee Scientific, Berkeley, CA). Real-time personal exposure to CO was measured with Lascar CO monitors (model EL-USB-300, Lascar Electronics, Erie, PA). Further details on equipment, exposure assessment procedures and data processing are reported elsewhere (Johnson et al., 2020). Additional follow-up home study visits occurred two additional times before birth, at 24–28 weeks and 32–36 weeks gestation where trained local field staff repeated many of the same procedures that occurred at the baseline visit. Adverse events and serious adverse events were defined according to standard definitions used in clinical trials (OHRP, 2008). Participants were monitored for adverse events and serious adverse events throughout the trial. Whenever an event occurred, the field staff collected detailed information on the appropriate case report form. Data were collected on password-protected tablets using the Research Electronic Data Capture (REDCap™) system (Harris, 2019, Harris et al., 2009).

The outcomes of spontaneous abortion, post-partum hemorrhage and hypertensive disorders of pregnancy were reported as adverse events. Spontaneous abortion (SAB) is defined as a loss of fetus before 20 weeks of gestation (calculated from gestational age at recruitment and confirmed by ultrasonography). Post-partum hemorrhage is defined as a loss of more than 500 ml to 1,000 ml of blood within the first 24 h following childbirth. The outcome of hypertensive disorders of pregnancy includes preeclampsia, defined as a new onset of hypertension and proteinuria or the new onset of hypertension and significant end-organ dysfunction with or without proteinuria after 20 weeks of gestation or postpartum in a previously normotensive woman. Hypertensive disorders of pregnancy also include cases of gestational hypertension defined as the new onset of hypertension (defined as systolic blood pressure ≥ 140 mmHg and/or diastolic blood pressure ≥ 90 mmHg) at ≥ 20 weeks of gestation. Participants reporting pre-existing hypertension were excluded from the hypertensive disorders of pregnancy outcomes analysis. Maternal mortality was reported as a serious adverse event defined as the death of a woman while pregnant or within 42 days of termination of pregnancy due to complications from pregnancy or childbirth but irrespective of the duration of the pregnancy.

### 2.5 Statistical analysis

Baseline data were first summarized by frequencies and percentages for categorical variables and by means and standard deviation (SD) for continuous variables; missing data were reported separately. Second, we compared outcomes using two sample t-tests for continuous variables and chi-square tests for categorical variables. Third, we conducted intention-to-treat (ITT) analyses according to the randomized allocation. Due to the small number of outcomes we did not include indicator variables to account for the stratification of the 10 randomized country sites in our model (four IRCs, with two randomization strata in India and six strata in Peru). Binary outcomes of SAB, postpartum hemorrhage, hypertensive disorders of pregnancy, and maternal mortality were compared between the intervention and control arms using log- binomial regression models. We performed two-tailed hypothesis tests at an α-level of 0.05 and calculated risk ratios for all binary outcomes. We also created a composite score by summing reported SAB, hypertensive disorders of pregnancy, postpartum hemorrhage and maternal mortality into one binary adverse neonatal and fetal outcome (yes/no). The composite score accounted for the potential for multiple outcomes in the same participant.

## 3 Results

### 3.1 Baseline characteristics

**Fig. 1:**
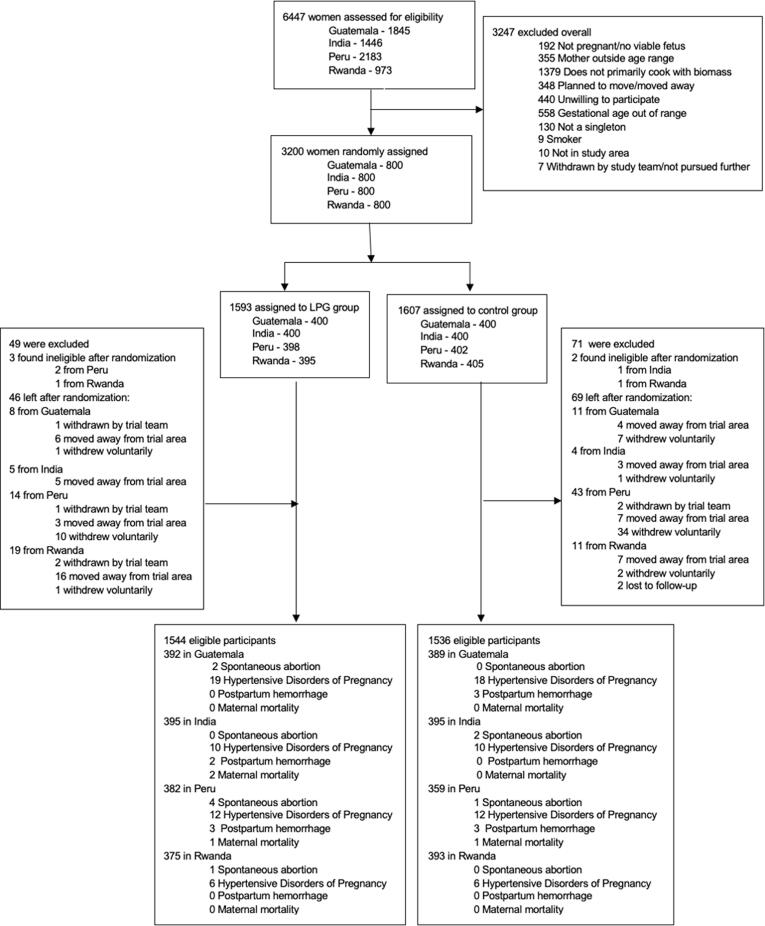
CONSORT flow chart.

Table 1 describes baseline characteristics of intervention versus control arms; no differences were observed. Randomization resulted in balanced arms. Five of the 3200 women were determined ineligible after randomization, resulting in 3195 total participants (1590 in the intervention arm and 1605 in the control arm) included in the analysis. Fig. 1 presents the Consolidated Standards of Reporting Trials (CONSORT) flow diagram (Moher et al., 2010). The mean gestational age at enrollment was 15.4 (SD 3.1) weeks overall and was balanced across study arms. Most of the women were in either the 20–24 (37.4%) or 25–29 (31.8%) age groups. Enrolled women were evenly distributed across educational categories. According to the Minimum Dietary Diversity for Women indicator, over half of the participants (56.2%) fell into the low category in the minimum dietary diversity score and only 11.5% were in the high dietary diversity category (FAO and FHI 360, 2016). Utilizing the Food Insecurity Experience Scale, over half reported being food secure (56.1%) and 15.4% experienced severe food insecurity (Ballard et al., 2013). The mean body mass index (BMI) for pregnant women at enrollment was 23.2 (SD 4.1) kg/m^2^ which falls into the normal weight category according to WHO BMI ranges (WHO, 2010). Overall, 38.4% were nulliparous and 13.2% reported a history of a previous spontaneous abortion.

**Table 1:** Demographic characteristics at baseline by control and intervention arm.

		Control n = 1605	Intervention n = 1590	Overall n = 3195^1^
*Maternal characteristics at baseline*				
Gestational week at baseline, mean (SD)		15.3 (3.2)	15.5 (3.1)	15.4 (3.1)
Maternal age, years, mean (SD)		25.4 (4.5)	25.3 (4.4)	25.4 (4.5)
	<20	209 (13.0%)	189 (11.9%)	398 (12.5%)
	20–24	579 (36.1%)	616 (38.7%)	1195 (37.4%)
	25–29	517 (32.2%)	500 (31.4%)	1017 (31.8%)
	30–35	300 (18.7%)	285 (17.9%)	585 (18.3%)
Highest level of education achieved, n (%)				
	No formal education	558 (34.8%)	481 (30.3%)	1039 (32.5%)
	Primary completed	533 (33.2%)	558 (35.1%)	1091 (34.1%)
	Secondary completed	514 (32.0%)	550 (34.6%)	1064 (33.3%)
	Missing	0 (0.0%)	1 (<0.1%)	1 (0.0%)
Minimum dietary diversity^2^, Category (score), n (%)				
	Low (<4)	906 (56.4%)	890 (56.0%)	1796 (56.2%)
	Medium (4–5)	533 (33.2%)	496 (31.2%)	1029 (32.2%)
	High (>5)	165 (10.3%)	203 (12.8%)	368 (11.5%)
	Missing	1 (0.1%)	1 (0.1%)	2 (0.1%)
Household food insecurity^3^, Category (score), n (%)				
	Food secure	863 (53.8%)	930 (58.5%)	1793 (56.1%)
	Mild (1, 2, 3)	448 (27.9%)	416 (26.2%)	864 (27.0%)
	Moderate (4, 5, 6) / Severe (7, 8)	272 (16.9%)	220 (13.8%)	492 (15.4%)
	Missing	22 (1.4%)	24 (1.5%)	46 (1.4%)
BMI (kg/m^2^), mean (SD); n missing		23.1 (4.0); 7	23.3 (4.1); 12	23.2 (4.1); 19
Mother’s hemoglobin level (g/dL), mean (SD); n missing		12.5 (1.9); 17	12.4 (1.9); 13	12.5 (1.9); 30
Reported vitamin intake^4^, n (%)				
Multiple micronutrient tablets		198 (12.3%)	181 (11.4%)	379 (11.9%)
Iron		974 (60.7%)	947 (59.6%)	1921 (60.1%)
Vitamin A		15 (0.9%)	10 (0.6%)	25 (0.8%)
Folate		911 (56.8%)	877 (55.2%)	1788 (56.0%)
Other		46 (2.9%)	44 (2.8%)	90 (2.8%)
None		314 (19.6%)	342 (21.5%)	656 (20.5%)
Nulliparous^5,^ n (%)				
Yes		589 (36.7%)	639 (40.2%)	1228 (38.4%)
No		1014 (63.2%)	947 (59.6%)	1961 (61.4%)
Missing		2 (0.1%)	4 (0.3%)	6 (0.2%)
History of spontaneous abortion, n (%)				
Yes		219 (13.6%)	203 (12.8%)	422 (13.2%)
No		1386 (86.4%)	1387 (87.2%)	2773 (86.8%)
*Exposure characteristics*				
Someone in household smokes^6^, n (%)				
Yes		181 (11.3%)	153 (9.6%)	334 (10.5%)
No		1421 (88.5%)	1436 (90.3%)	2857 (89.4%)
Missing		3 (0.2%)	1 (<0.1%)	4 (0.1%)
Mean personal 24-hr exposure (SD)				
Particulate Matter (PM_2.5_) (µg/m3)		111 (1 1 0)	120 (1 3 5)	115 (1 2 3)
Black Carbon (µg/m^3^)		12.4 (9.4)	12.6 (11.0)	12.5 (10.2)
Carbon Monoxide (ppm)		2.3 (4.0)	2.7 (4.8)	2.5 (4.4)
*Household characteristics*				
Number of people sleeping in house, mean (SD); n missing		4.3 (2.0); 0	4.3 (2.0); 1	4.3 (2.0); 1
Owns household assets, n (%)				
	Color Television	783 (48.8%)	774 (48.7%)	1,557 (48.7%)
	Radio	721 (44.9%)	734 (46.2%)	1,455 (45.5%)
	Mobile phone	1,395 (86.9%)	1,388 (87.3%)	2,783 (87.1%)
	Bicycle	409 (25.5%)	365 (23.0%)	774 (24.2%)
	Bank account	628 (39.1%)	697 (43.8%)	1,325 (41.5%)

^1^N = 3,200 women were randomized; 5 women were deemed ineligible after randomization ^2^Adapted from Food and Agriculture Organization of the United Nations Minimum Diet Diversity for Women (FAO and FHI 360, 2016). ^3^The Food Insecurity Experience Scale, developed by the Food and Agriculture Organization of the United Nations (Ha et al., 2018) ^4^ Vitamins reportedly taken in the past 12 months.

^5^Nulliparous defined as zero pregnancies reaching 20 weeks and 0 days of gestation or beyond; miscarriages can have occurred in a woman who is nulliparous.

^6^Someone in the household other than the pregnant woman smokes; pregnant women were all non-smokers based on eligibility criteria.

Enrolled pregnant women reported iron (69.1%) and folate (56.0%) supplementation, and the overall mean hemoglobin was 12.5 g/dL (SD 1.9). The mean number of people sleeping in a household was 4.3 (SD 2.0). In terms of household assets, 87.1% of participant households owned a mobile phone, 41.5% had a bank account, 48.7% had a color television and 45.5% had a radio. Baseline PM_2.5_, BC, and CO reported as arithmetic means were similar between intervention and control arms. To meet inclusion criteria, enrolled women were non-smokers, but 10.5% reported a smoker in their household.

### 3.2 Maternal outcomes

**Fig. 2:**
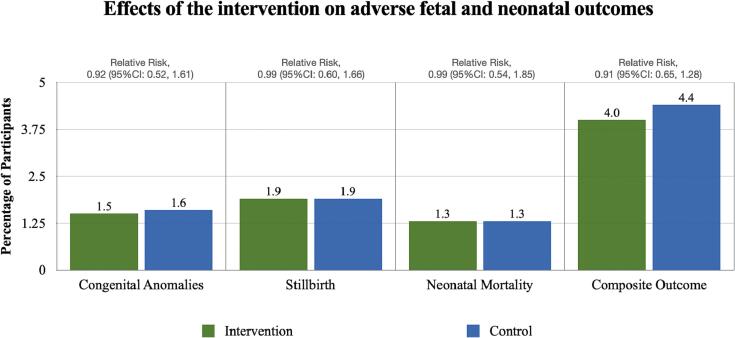
Effects of the intervention on adverse maternal outcomes. Note: Y axis range is limited to10% of participants in order to visualize the results.

Among the 3200 women enrolled in the study, 3080 remained eligible to be included in the analysis of adverse maternal outcomes. Women with preexisting hypertension were also excluded from the analysis of the hypertensive disorders of pregnancy outcome (n = 18). Maternal outcomes by study arm are presented in Table 2 and Fig. 2. There were 10 SAB (7 intervention, 3 control), 93 hypertensive disorders of pregnancy (47 intervention, 46 control), 11 post postpartum hemorrhage (5 intervention, 6 control), and 4 maternal deaths (3 intervention, 1 control). The overall incidence of SAB was 0.5% in the intervention arm and 0.2% in the control arm. Compared to the control arm, the relative risk of SAB among women in the intervention arm was 2.32 (95% CI: 0.60, 8.96). The overall incidence of hypertensive disorders of pregnancy was 3.0% in the intervention arm and 3.0% in the control arm. Compared with the control arm, the relative risk of hypertensive disorders of pregnancy among women in the intervention arm was 1.02 (95% CI: 0.68, 1.52). The overall incidence of postpartum hemorrhage was 0.3% in the intervention arm and 0.4% in the control arm. Compared with the control arm, the relative risk of postpartum hemorrhage among women in the intervention arm was 0.83 (95% CI: 0.25, 2.71). The overall incidence of maternal mortality was 0.2% in the intervention arm and 0.1% in the control arm. Compared with the control arm, the relative risk of maternal mortality among women in the intervention arm was 2.98 (95% CI: 0.31, 28.66). Finally, the proportion of overall adverse maternal composite outcome (SAB, hypertensive disorders of pregnancy, postpartum hemorrhage, and maternal mortality) was 4.0% in the intervention arm and 3.6% in the control arm. Compared with the control arm, the relative risk of a composite adverse maternal outcome among women randomized to the intervention was 1.10 (95% CI: 0.77, 1.58). All 95% CIs for the relative risk include the null value of 1 and suggest insufficient evidence that the groups are statistically significantly different. Table A1 displays results of maternal outcomes by study arm stratified by each research center.

**Table 2:** Effects of the intervention on spontaneous abortion, hypertensive disorders of pregnancy, postpartum hemorrhage, maternal mortality and composite outcome.^1^

**Outcome**	**Intervention** ***n (%)***	**Control** ***n (%)***	**Relative Risk**^2^ ***(95% CI)***
Spontaneous abortion			
Yes	7 (0.5)	3 (0.2)	2.32 (0.60, 8.96)
No	1537 (99.5)	1533 (99.8)	
Hypertensive disorders of pregnancy			
Yes	47 (3.0)	46 (3.0)	1.02 (0.68, 1.52)
No	1488 (97.0)	1481 (97.0)	
Postpartum hemorrhage			
Yes	5 (0.3)	6 (0.4)	0.83 (0.25, 2.71)
No	1539 (99.7)	1530 (99.6)	
Maternal mortality			
Yes	3 (0.2)	1 (0.1)	2.98 (0.31, 28.66)
No	1541 (99.8)	1535 (99.9)	
Composite outcome			
Yes	61 (4.0)	55 (3.6)	1.10 (0.77, 1.58)
No	1474 (96.0)	1472 (96.4)	

1 Denominator for spontaneous abortion, postpartum hemorrhage, maternal mortality was 3080. Deonominator for hypertensive disorders of pregnancy and composite was 3062.

2 Relative risk reported as intervention compared to control.

## 4 Discussion

In this trial conducted in four diverse LMIC locations we found that adverse maternal outcomes (spontaneous abortion, hypertensive disorders of pregnancy, postpartum hemorrhage and maternal mortality) did not differ based on randomization group. The LPG intervention demonstrated a reduction in exposure to fine particulate matter, carbon monoxide and black carbon, yet the LPG intervention did not lower the risk of maternal outcomes included in this study (Johnson, et al., 2021). Also, with high levels of fidelity and adherence our null findings do not seem to be driven by issues with compliance with the intervention (Quinn et al., 2021). The LPG intervention may not have been introduced early enough in pregnancy to have a measurable effect. Enrollment to the HAPIN trial and introduction of the LPG intervention mainly occurred during the second trimester and we may have missed important windows of gestational development that are more sensitive to household air pollution exposure. While the study protocol allowed for enrollment as early as 9 weeks gestation, the mean enrollment of participants in the second trimester may be attributed to delayed access to early antenatal care as well as issues around scheduling enrollment ultrasounds to ensure gestational age accuracy. Prior research supports the association between chronic exposure to air pollution and adverse pregnancy outcomes. Mukherjee et al. (2015) investigated the effects of cumulative biomass smoke exposure on adverse reproductive outcomes by measuring PM_10_ concentrations in kitchens of premenopausal women in rural India who exclusively cooked with biomass fuels over the past 5 years. Compared to women who used LPG stoves, lifetime exposure to biomass smoke increased the odds of spontaneous abortion (Mukherjee et al., 2015). Also, in a US-based prospective cohort study among couples trying to conceive, Ha et al. (2018) reported an increase in pregnancy loss per interquartile increase of ambient PM_2.5_ across the entire pregnancy (hazard ratio (HR) 1.13, 95% CI: 1.03, 1.24). The authors concluded that chronic exposure may be more detrimental to pregnancy loss than acute exposure during specific gestational windows (Dugas et al., 2022). Future research examining the effects of exposure to household air pollution on adverse pregnancy outcomes before conception and throughout the entire pregnancy is needed. Our findings are consistent with the only other RCT that included adverse maternal outcomes, including spontaneous abortion. The trial randomized 324 pregnant women in urban Nigeria to either continue cooking with kerosene/firewood stove (control) or use an ethanol stove (intervention). With only 4 reported SABs, while fewer occurred in the ethanol group (1 ethanol, 3 control), the authors concluded the numbers were too small to uncover significant differences between groups. Since approximately 80% of early pregnancy loss occurs in the first trimester, SAB outcomes in our study may also have been under-reported since the mean gestational age of enrollment for the HAPIN trial occurred at 15.4 (SD 3.1) weeks (Dugas and Slane, 2022). There are several limitations to this study. The outcomes in this study were exploratory outcomes within the larger HAPIN trial. The trial sample size was based on power calculations for the primary outcomes of the HAPIN trial and may not have been sufficient to detect differences in rarer outcomes of adverse pregnancy outcomes presented in this study. Due to the low numbers, we could not evaluate subgroups by gestational age at intervention. Second trimester enrollment also limited our estimate of SAB outcomes that more frequently occur in the first trimester. Also, recruitment occurred at prenatal clinics and we thus did not recruit pregnant women who may not have access to or do not seek antenatal care. Finally, since we restricted our exposure measurements to particulate matter, black carbon and carbon monoxide, we may have missed other pollutants emitted by the LPG stoves that may have detrimental health effects rendering the intervention insufficient to impact health outcomes. This study also has several strengths. The HAPIN trial was the largest, and the first, multi-country RCT to use a standardized protocol to assess the effect of a LPG stove intervention on exposure to household air pollution and adverse pregnancy outcomes. Personal exposure measurements of PM_2.5_, black carbon and carbon monoxide during pregnancy, coupled with stove use monitoring to assess stove stacking, enabled comprehensive assessments of household air pollution (Johnson et al., 2021). Even during the Covid-19 pandemic, field staff, participants and researchers continued to conduct the trial resulting in low loss to follow-up and high fidelity and adherence to the intervention (Quinn et al., 2021). This study addressed many of the gaps in the literature and was the first study to explore household air pollution exposure on multiple maternal outcomes in LMICs.

## 5 Conclusions

The HAPIN trial was the first multi-country RCT of LPG stove and fuel distribution in nearly 3200 households; forthcoming papers will examine intervention effects on child stunting and pneumonia during infancy, and a range of secondary outcomes including early child development. We did not observe a statistically significant reduction in incidence of adverse maternal outcomes within the LPG intervention arm compared to women in the control arm. These results do not support a beneficial effect of the intervention on these outcomes despite evidence that the LPG intervention reduced household air pollution exposure and achieved high fidelity and adherence by the trial participants (Quinn et al., 2021, Johnson, et al., 2021). The mechanisms behind adverse maternal outcomes are multifactorial and can include genetics, demographics, past medical history, and access to good prenatal care, but many cases have unknown etiologies. While the LPG intervention reduced household air pollution exposure substantially, it may not have been introduced early enough in gestation to make an impact on adverse maternal outcomes.

**Author Contributions:** Conceptualization, Ashley Younger, Rachel Craik, Thomas Clasen and Lisa M. Thompson; Data curation, Lisa Elon, Jiantong Wang and Howard H. Chang; Formal analysis, Ashley Younger, Abbey Alkon, Wenlu Ye, Howard H. Chang, Wenlu Ye, Thomas Clasen and Lisa M. Thompson; Investigation, Anaité Diaz-Artiga, John McCracken, Adly Castañaza Gonzalez, Libny Monroy Alarcon, Kalpana Balakrishnan, Ashley Toenjes and Lisa M. Thompson; Methodology, Ashley Younger, Abbey Alkon, Kristen Harknett, Miles Kirby, Amy Lovvorn, Wenlu Ye, Ajay Pillarisetti, Aris Papageorghiou, Ashlinn Quinn, Howard H. Chang, Wenlu Ye, Jennifer Peel and Lisa M. Thompson; Project administration, Amy Lovvorn, Anaité Diaz-Artiga, John McCracken, Adly Castañaza Gonzalez, Libny Monroy Alarcon, Alexie Mukeshimana, Ghislaine Rosa, Marilu Chiang, Kalpana Balakrishnan, Sarada Garg, Ajay Pillarisetti, Ricardo Piedrahita, Michael Johnson, Rachel Craik, Kendra N. Williams, Lindsay Underhill, William Checkley, Jennifer Peel, Thomas Clasen and Lisa M. Thompson; Supervision, Abbey Alkon, Kristen Harknett, Anaité Diaz-Artiga, John McCracken, Ghislaine Rosa, Kalpana Balakrishnan, Michael Johnson, Aris Papageorghiou, Kendra N. Williams, Lindsay Underhill, Howard H. Chang, Luke Naeher, Joshua Rosenthal, William Checkley, Jennifer Peel, Thomas Clasen and Lisa M. Thompson; Writing – original draft, Ashley Younger; Writing – review & editing, Abbey Alkon, Kristen Harknett, Miles Kirby, Lisa Elon, Amy Lovvorn, Wenlu Ye, Anaité Diaz-Artiga, John McCracken, Kalpana Balakrishnan, Sarada Garg, Ricardo Piedrahita, Michael Johnson, Rachel Craik, Aris Papageorghiou, Ashley Toenjes, Ashlinn Quinn, Kendra N. Williams, Lindsay Underhill, Luke Naeher, Joshua Rosenthal, William Checkley, Wenlu Ye, Jennifer Peel, Thomas Clasen and Lisa M. Thompson.

**Funding:** This study is funded by the U.S. National Institutes of Health (NIH; cooperative agreement 1UM1HL134590) in collaboration with the Bill & Melinda Gates Foundation (OPP1131279). Participating NIH organizations include the National Heart, Lung and Blood Institute, National Institute of Environmental Health Sciences, National Cancer Institute, National Institute of Child Health and Human Development, Fogarty International Center, and the NIH Common Fund.

**Institutional Review Board Statement:** The consent forms and study protocol were reviewed and approved by institutional review boards (IRBs) or ethics committees at Emory University (00089799), Johns Hopkins University (00007403), Sri Ramachandra Institute of Higher Education and Research (IEC- N1/16/JUL/54/49) and the Indian Council of Medical Research– Health Ministry Screening Committee [5/8/4–30/(Env)/Indo-US/ 2016-NCD-I], Universidad del Valle de Guatemala (146–08-2016/ 11–2016) and Guatemalan Ministry of Health National Ethics Committee (11–2016), Asociacion Benefica PRISMA (CE2981.17), London School of Hygiene and Tropical Medicine (11664–5), Rwandan National Ethics Committee (No. 357/RNEC/2018), and Washington University in St. Louis (201611159).

**Informed Consent Statement:** Informed consent was obtained from all study participants that met eligibility requirements using standard procedures. Participants were able to withdraw from the study at any time.

## Declaration of Competing Interest

The authors declare that they have no known competing financial interests or personal relationships that could have appeared to influence the work reported in this paper.

## Figures and Tables

**Table A1:** Spontaneous abortion, hypertensive disorders of pregnancy, postpartum hemorrhage, maternal mortality and composite outcome by country site, n(%).

	**Guatemala**		**India**		**Peru**		**Rwanda**	
	**I**	**C**	**I**	**C**	**I**	**C**	**I**	**C**
Spontaneous abortion								
Yes	2 (0.5)	0 (0.0)	0 (0)	2 (0.5)	4 (1.0)	1 (0.3)	1 (0.3)	0 (0.0)
No	390 (99.5)	389 (100.0)	395 (100.0)	393 (99.5)	378 (99.0)	358 (99.7)	374 (99.7)	393 (100.0)
Hypertensive disorders of pregnancy								
Yes	19 (4.9)	18 (4.7)	10 (2.5)	10 (2.5)	12 (3.2)	12 (3.4)	6 (1.6)	6 (1.5)
No	370 (95.1)	367 (95.3)	385 (97.5)	385 (97.5)	368 (96.8)	346 (96.6)	365 (98.4)	383 (98.5)
Postpartum hemmorhage								
Yes	0 (0.0)	3 (0.8)	2 (0.5)	0 (0.0)	3 (0.8)	3 (0.8)	0 (0.0)	0 (0.0)
No	392 (100.0)	386 (99.2)	393 (99.5)	395 (100.0)	379 (99.2)	356 (99.2)	375 (100.0)	393 (100.0)
Maternal Mortality								
Yes	0 (0.0)	0 (0.0)	2 (0.5)	0 (0.0)	1 (0.3)	1 (0.3)	0 (0.0)	0 (0.0)
No	392 (100.0)	389 (100.0)	393 (99.5)	395 (100.0)	381 (99.7)	358 (99.7)	375 (100.0)	393 (100.0)
Composite outcome								
Yes	21 (5.4)	21 (5.5)	13 (3.3)	12 (3.0)	20 (5.3)	16 (4.5)	7 (1.9)	6 (1.5)
No	368 (94.6)	364 (94.5)	382 (96.7)	383 (97.0)	360 (94.7)	342 (95.5)	364 (98.1)	383 (98.5)

## Data Availability

Data will be made available on request.

## Appendix A

Table A1
